# Unexpected combination of acute croup and myocarditis: Case report

**DOI:** 10.1186/1472-6890-5-5

**Published:** 2005-06-07

**Authors:** George Briassoulis, Athina Tsorva, Emmanuel Agapitos, John Papadatos

**Affiliations:** 1 Pediatric Intensive Care Unit, "P & A Kyriakou" Children's Hospital, Athens, Greece; 2 Paediatric Intensive Care Unit, University Hospital of Heraklion, Crete, Greece; 3 Microbiology and Transfusion Departments, NIMTS Hospital, Athens, Greece; 4 Department of Pathology, School of Medicine, National University of Athens, Greece

## Abstract

**Background:**

Lower vaccination coverage among foreign-born children is of concern because they live in households and communities characterized by more intense exposure to infectious diseases. Because of their higher prevalence rates, there is an increasing occurrence of infectious diseases imported into developed countries. This case report emphasizes the emerging necessity for new clinicians and pathologists of having competence with old infectious disease pathology.

**Case presentation:**

A three and a half year old girl, who presented with croup history of 5 days and has been in severe respiratory distress, was admitted to the Pediatric Intensive Care Unit in shock and acute respiratory failure. The patient was immediately intubated, and a grayish nonadherent membrane extending through the glottis down into the larynx was apparent during the procedure. Echocardiographic findings, which were consistent with acute myocarditis, confirmed poor left ventricular contractility despite escalating high doses of inotropes. Autopsy showed numerous strains of toxigenic corynobacterium diphtheriae, which also grew on the Loeffler cultures of membranes received during the intubation.

**Conclusion:**

It is critical that new generations of clinicians and bio-pathologists not only be trained in the subspecialty of infectious disease pathology, but that they also be willing participants in the diagnosis and investigation of infectious diseases.

## Background

Diphtheria, caused by *corynebacterium diiphtheriae*, is acquired by contact with either a carrier or a person with the disease. Before the discovery of antitoxin at the turn of the 20^th ^century, the "straggling angel of children", as diphtheria once was called, was a significant cause of mortality in children and adults. In severe cases, respiratory and circulatory collapse may occur. Complications secondary to elaborated diphtheritic toxin may affect any system, but myocarditis and nervous system involvement are most characteristic. The incidence of diphtheria declined markedly after the extensive use of diphtheria toxoid after World War II.

Inadequate immunization cover is deemed responsible for the continued menace of diphtheria in developing countries [1]. A large number of immigrants have come to Greece and other western countries from diphtheria-endemic countries during the past 10 years. Lower vaccination coverage among foreign-born children is of concern because foreign-born children often live in households and communities characterized by more intense exposure to infectious diseases, and many originate from countries with much higher prevalence rates of diphtheria and other diseases than the developed countries [2]. Additionally, the majority (65%) of internationally adopted children have no written records of overseas immunizations [3].

The increasing occurrence of infectious diseases imported into the United States and other nations, including human immunodeficiency virus-1 group O, dengue fever, tuberculosis, malaria, diphtheria and cholera in immigrants and travelers, and Ebola virus in nonhuman primates, emphasizes the necessity for clinicians and pathologists of having competence with infectious disease pathology [4]. It is critical that new generations of intensivists and bio-pathologists not only be trained in the subspecialty of infectious disease pathology, but that they also be willing participants in the diagnosis and investigation of infectious diseases. We describe a case of acute diphtheritic laryngotracheobronchitis and myocarditis in an immigrant girl, the first diagnosed in Greece during the last 30 years.

## Case presentation

**Figure 1:**
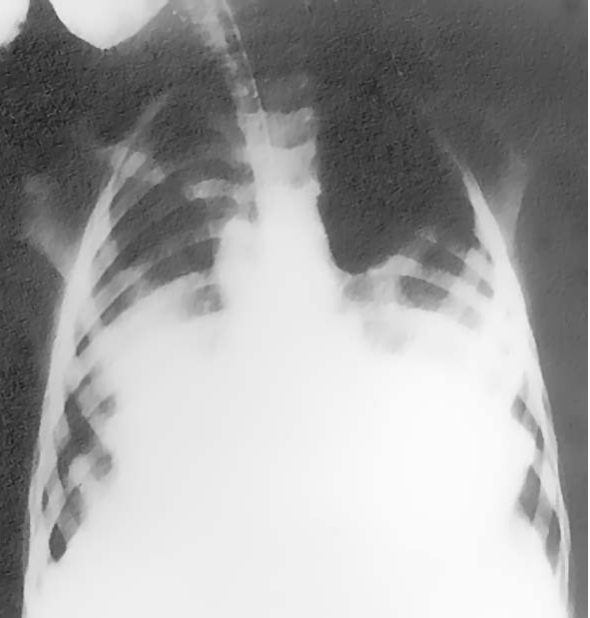
**Chest roentgenogram upon admission. **Frontal chest roentgenogram revealed extensive bilateral diffuse pulmonary infiltrations, left greater than right.

A three and a half year old girl, who presented with a croup history of 4 days duration, was transferred to the Pediatric Intensive Care Unit because of worsening respiratory distress. The past history was uncontributory except that the patient was an unvaccinated immigrant, living in close contact with her alcoholic grandfather. The patient was immediately intubated, and a grayish nonadherent membrane extending through the glottis down into the larynx was apparent during the procedure. Material from the membranes was sent for cultures and the patient was started on intravenous penicillin and specific antitoxin. Although blood gases improved initially, respirator settings had soon to be increased significantly to sustain a pO_2 _value above 60 mm Hg (7.9 kPa). A chest roentgenogram revealed extensive bilateral diffuse pulmonary infiltrations (Fig. 1). Laboratory findings included white blood cells 70,110 cu mm (70*10^9^/L) with segmented neutrophils 46%, band forms 8%, platelets 134,000 cu mm (134*10^9^/L), CRP 177. Serum alanine aminotransferase and aspartate aminotransferase concentrations peaked at 340 U/L and 721 U/L, respectively and serum creatinine increased to 1.4 mg/dL (124 μmol/L). On hospital day 1 she became hypotensive and remained in shock despite escalating doses of inotropic agents. Echocardiographic findings, which were consistent with acute cardiomyopathy, confirmed extremely poor left ventricular contractility (asystole; shortening fraction <5%), despite high dose continuous dobutamine (20 μg.kg-1.min-1) and epinephrine (up to 5 μg.kg-1.min-1) infusions. After having completed 36 hours of hospitalization in the PICU with multiple cardiac arrests, the child was no more responding to resuscitation efforts and died.

**Figure 2:**
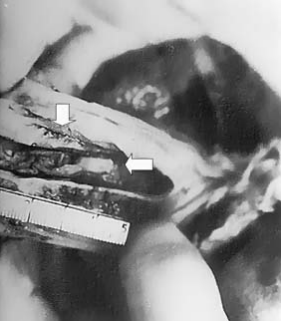
**Tracheobronchial membranes at autopsy. **Autopsy findings revealed numerous diphtheritic membrane formations (arrowheads) extended down into the larynx and further obstructing the distal tracheobronchial tree.

**Figure 3:**
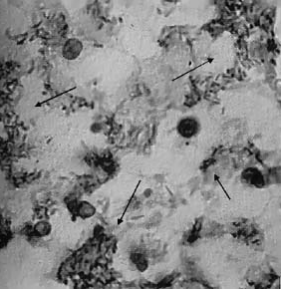
**Strains of corynobacterium diphtheriae on a smear. **A Giemsa stain (original magnification × 1000) of visceral hemorrhagic necrotic lesions was associated with numerous strains of corynobacterium diphthetiae, producing the typical club-shaped appearance and distributed in a typical Chinese-letter appearance on the smear (arrows).

Autopsy findings revealed numerous diphtheritic membrane formations extended down into the larynx and further obstructing the distal tracheobronchial tree (Fig. 2). Myocardium edema, congestion and mononuclear and neutrophil cell infiltration, completed the clinicopathologic picture of diphtheritic cardiomyopathy. Membrane formations and visceral hemorrhagic necrotic lesions were associated with numerous strains of corynobacterium diphtheriae (producing the typical club-shaped appearance and distributed in a typical Chinese-letter appearance on the smear) (Fig. 3). The organism, however, of Gravis type, grew on the Loeffler cultures of portions of membranes, which were received during the intubation. As also expected, the strain was shown to be toxigenic.

## Conclusion

This manuscript reports on an exceptionally rare disease – almost forgotten today – in a western country. Most of the recent literature concerning clinical manifestations and treatment modalities of diphtheritic croup [5,6] or myocarditis [7,8] almost exclusively comes from endemic areas in developing countries [9,10]. Recently, however, concerns regarding the emergence of this obsolete disease in the developed word are reported with an increasing frequency, mostly as quizzes [11] exceptional [12] or unusual case reports [13,14] – like ours – or as alarming updated review articles of progress in clinical, epidemiological and microbiological aspects of diphtheria in the European region [15]. Researchers concern with the emerging threat of diphtheria being re-introduced from Eastern Europe or Asia and re-established in the West, highlight the need for improved immunisation coverage, surveillance and epidemiological studies to sustain control of diphtheria in European Region [16], and overemphasize the need for keeping a high index of clinical suspicion and initiation treatment without delay [17].

Airway obstruction is the most common cause of death among culture-confirmed cases of diphtheria involving the respiratory tract [18]. Although diphtheritic membranes in the larynx, dyspnea and leukocytosis are all poor prognostic signs, tracheobronchial membrane is not usually identified before death in patients with respiratory tract diphtheria. In a recent study examining clinical features and predictors of diphtheritic cardiomyopathy in Vietnamese children, it was shown that fatal outcome was best predicted by the combination of myocarditis on admission and a pseudomembrane score of >2 [7]. Similarly, in Indian children, the extension of membrane formation to two or more sites was a highly sensitive predictor of mortality, whereas a total leukocyte count > 25,000 cu mm (25*10^9^/L) had a high specificity and positive predictive value while serum alanine aminotransferase levels of > 80 U/L had high sensitivity and a negative predictive value [19], findings which were verified in our case report. Cardiomyopathy and neuropathy occur frequently, complicating severe diphtheria, but in most instances the cardiac manifestations appear between the second and the third week of the disease. Occasionally, myocarditis, as in our case, and neuritis may be noted as early as the first week of the disease [20]. Because diphtheritic antitoxin cannot neutralize penetrating toxin or toxin absorbed to cells, its efficacy decreases as the duration of pharyngeal diphtheria increases. Therefore, the late provision of specific treatment after the 4^th ^day is associated with a 20-fold increase in mortality rate [21]. Nowadays, reports from endemic areas offer new scientific information on current treatment modalities for obsolete diseases. A Russian study showed that patients with severe diphtheritic myocarditis had a high risk of lowering of left ventricular ejection fraction below 35%, akinesia of left ventricular segments (asinergia index > 2) and death [22]. Interestingly, temporary insertion of a cardiac pacemaker in these patients has been shown to improve outcome [23].

Diphtheria caused by the gravis strain is usually more toxigenic and carries a poorer prognosis. Although adequate immunization rate has been suggested to result in a form of herd immunity by dampening the survival of toxigenic strains of corynebacterium diphtheriae, the gene for virulence can be transferred by lysogeny to nontoxigenic strains to confer the toxigenic state among people who are not immune [24].

Diphtheria has been regarded eliminated in Greece like in most Western European countries after the start of mass immunizations 3 decades ago, and no indigenous case was reported since 1970s. Meanwhile, it seems that immunologic status of population has been changed. Most recent diphtheria outbreaks in western countries have increasingly involved alcoholic urban adults and cutaneous infection has been recognized as an important epidemiologic feature of such modern outbreaks [25]. That accorded with the continuing decline of diphtheria antitoxin titter after vaccination with protective levels (>0.1 IU/mL) detected in only 15% of those more than 40 years of age [26]. Thus, the effect of massive vaccination routines is of particular concern now, because, although the incidence of diphtheria in general has been decreased, the mortality from toxic diphtheria remains high, especially between unvaccinated persons [27]. For that reason, regular booster doses with the combined tetanus-diphtheria toxoid should be routinely given at mid-decade ages and whenever tetanus toxoid is indicated. Moreover, since it is more than obvious now that immigration of nonimmunized, impoverished populations has already provided a continuing pool of susceptible persons, prompt massive immunization campaign should be urgently aimed at such unimmunized groups.

## Competing interests

The author(s) declare that they have no competing interests.

## Authors' contributions

GB conceived of the study, participated in its design and coordination, and drafted the manuscript. JP has made substantial contributions to conception. TA helped to draft the manuscript. EA participated in the technical procedures, collection of tissue samples, immunohistochemistry, special staining and cultures. All authors read and approved the final manuscript.

## Pre-publication history

The pre-publication history for this paper can be accessed here:


